# Control of Dopant Distribution in Yttrium-Doped Bioactive Glass for Selective Internal Radiotherapy Applications Using Spray Pyrolysis

**DOI:** 10.3390/ma12060986

**Published:** 2019-03-25

**Authors:** Abadi Hadush Tesfay, Yu-Jen Chou, Cheng-Yan Tan, Fetene Fufa Bakare, Nien-Ti Tsou, E-Wen Huang, Shao-Ju Shih

**Affiliations:** 1 Department of Materials Science and Engineering, National Taiwan University of Science and Technology, Taipei 106, Taiwan; d10504802@mail.ntust.edu.tw (A.H.T.); m10604317@mail.ntust.edu.tw (C.-Y.T.); d10504825@mail.ntust.edu.tw (F.F.B.); 2 Department of Mechanical Engineering, National Taiwan University of Science and Technology, Taipei 106, Taiwan; yu-jen.chou@mail.ntust.edu.tw; 3 Department of Materials Science and Engineering, National Chiao Tung University, No. 1001, Tahsueh Road, HsinChu 300, Taiwan; tsounienti@nctu.edu.tw (N.-T.T.); ewhuang@g2.nctu.edu.tw (E.-W.H.)

**Keywords:** bioactive glass, Yttrium, electron microscopy, spray pyrolysis

## Abstract

In this study, we demonstrate the fabrication of Y-doped bioactive glass (BG), which is proposed as a potential material for selective internal radiotherapy applications. Owing to its superior bioactivity and biodegradability, it overcomes the problem of yttrium aluminosilicate spheres that remain in the host body for a long duration after treatment. The preparation of Y-doped BG powders were carried out using a spray pyrolysis method. By using two different yttrium sources, we examine the change of the local distribution of yttrium concentration. In addition, characterizations of phase information, particle morphologies, surface areas, and bioactivity were also performed. The results show that both Y-doped BG powders are bioactive and the local Y distribution can be controlled.

## 1. Introduction

Bioactive glass (BG) has been studied extensively since its first report by Hench et al. [1]. It was the first synthetic material to show a bone-bonding ability and has been successfully applied to various applications such as drug carriers, tooth fillers, and bone scaffolds [2,3,4]. Among these three applications, the development of drug carriers was the latest and is not mature yet. Therefore, in recent years, one popular application of drug carrier BG is selective internal radiation therapy (SIRT), which has been used for tumor tissue treatments [5,6,7].

The SIRT therapy is limited by the need to employ low doses of radiation to reduce damage to healthy tissues near the affected area [8], and so far the most common and popular material is yttrium aluminosilcate (YAS, Y_2_O_3_-Al_2_O_3_-SiO_2_) spheres [9,10]. The therapeutic use of Y microspheres was first prepared by Ehrhard et al. [11] and it has been used for more than 20 years owing to its advantage of high chemical stability and short half-life [12]. Since then, studies of Fe-doped, Tm-doped, and Er-doped YAS were reported [13,14]. However, these spheres have some serious drawbacks that need to be overcome; first, the YAS spheres are not biodegradable, and therefore, it will reside in the host for a long time and may cause damage after the end of the radioactive treatments [15]; second, the YAS spheres are biologically inactive and therefore do not establish a favorable interaction with the treated area [5]. Thus, Y-doped BG powders offer the merits of biodegradability and strong interaction with the treated tissues to become one of the potential candidates for SIRT applications [16]. 

Although Y-doped BG powders have been applied for radioisotope vectors, only a few studies have been reported [5,6,7]. For example, Cacaina et al. prepared the Y-doped BG powders as radioisotope vectors using conventional glass melting process and studied its behavior in simulated body fluid (SBF) [17]. Since the ^90^Y isotope is the main radioactive source to kill tumors in the Y-doped BG powder, the position of Y in BGs has started to attract some attention: First, the environment of Y in BG has been studied by Christie et al. [6]. Then, in 2015, Tilocca built the ion exchange model to use a molecular dynamics simulation to discuss the structural configuration of Y in a BG matrix to influence the biodegrading properties [5]. Though Y distribution in BG has been studied by the above simulation works with the assumption of a homogenous distribution for Y [5,6], according to our previous studies, Y distribution of concentrations may be inhomogeneous and strongly correlates with the experimental parameters (e.g., heat treatment conditions [18] and precursor properties [19]). Since the β radiation emitted by Y has low penetrating power, for better radiation efficiency, it is essential to control the Y distribution. Therefore, in this study, Y-doped BG powders were prepared from two common Y precursors such as yttrium acetate (YAc) and yttrium nitrate (YN).

In this study, pure BG powder and two Y-doped BG powders from YAc and YN salt were synthesized using spray pyrolysis (SP). Then, phase compositions, surface morphologies, inner structures, chemical compositions, and specific surface areas of powders were obtained using X-ray diffraction (XRD), scanning electron microscopy (SEM), focus ion beam (FIB), energy-dispersive X-ray spectroscopy (EDS), and the Brunauer–Emmet–Teller (BET) method, respectively. In addition, the local Y concentrations were obtained using several cross-sectional Y-doped BG particles. Finally, the bioactive tests were carried out and characterized using a Fourier transform infrared reflection (FTIR) spectrophotometer.

## 2. Materials and Methods

### 2.1. Specimen Preparation

First, pure BG powder was synthesized using spray pyrolysis based on the composition of 58S (60 mol% SiO_2_, 35 mol% CaO, and 5 mol% P_2_O_5_). The precursor solution was prepared by adding tetraethyl orthosilicate (TEOS, Si(OC_2_H_5_)_4_, 99.9 wt%, Showa, Tokyo, Japan), calcium nitrate tetrahydrate (CN, Ca(NO_3_)_2_·4H_2_O, 98.5 wt%, Showa, Tokyo, Japan), and triethyl phosphate (TEP, (C_2_H_5_)_3_PO_4_, 99.0 wt%, Alfa Aesar, Heysham, UK) into 60.00 g of ethanol with 1.60 g of 0.5 M diluted hydrochloric acid. The solution was then stirred at room temperature for 2 h to reach homogeneity. In addition, the precursor solutions of Y-doped BG powders were prepared by mixing an additional 10 wt% of yttrium source, either yttrium acetate (YAc, YC_6_H_9_O_6_, Sigma Aldrich, St. Louis, MO, USA) or yttrium nitrate (YN, YNO_3_O_9_∙6H_2_O, 99.8%, Sigma Aldrich, St. Louis, MO, USA), into the pure precursor solution and stirred for 2 h as well. For the spray pyrolysis process, all precursor solutions were poured into an ultrasonic atomizer (KT-100A, King Ultrasonic, New Taipei, Taiwan) operating at a frequency of 1.67 MHz. The atomized droplets were led into a tube furnace (D110, Dengyng, New Taipei, Taiwan) with three different heating zones. The temperature of each zone was set at 250 °C, 550 °C, and 300 °C for preheating, calcination, and cooling, respectively. At the exit of the furnace, a high voltage of 16 kV was applied to charge the surface of the synthesized powders. The charged powders were then neutralized and condensed inside an earthed stainless steel electrostatic collector.

### 2.2. Characterization

First, an X-ray diffractometer (XRD, D2 Phaser, Bruker, Karlsruhe, Germany) with Cu-Kα radiation was used to obtain the phase compositions using a collection angle that ranged from 20° to 80°. Next, the morphologies of all BG particles were examined using a scanning electron microscope (SEM, JSM-6500F, JEOL, Tokyo, Japan). To get reliable average particle sizes, more than 300 particles were measured from several SEM images for each specimen. In addition, a constant-volume adsorption apparatus (Novatouch LX2, Quantachrome Instruments, Boynton Beach, FL, USA) was operated at −196 °C to obtain nitrogen adsorption and desorption isotherms. The specific surface areas of pure BG powder and Y-doped BG powders were computed using the BET method. At last, for the characterization of inner structure and Y distributions, the cross-section specimens were prepared by focused ion beam (FIB, Quanta 3D FEG, FEI, Hillsboro, OR, USA) and the atomic compositions of each specimen were examined using energy dispersive spectroscopy (EDS, Oxford Instruments, Abingdon, UK) analysis.

### 2.3. In Vitro Bioactivity Test

The in vitro bioactivity tests of all BG powders were carried out using the SBF which has an ionic concentration similar to human plasma [20]. The test specimens were prepared by immersing the powders in SBF solution a solid to liquid ratio of 1 mg to 5 mL and were held at 37 °C for 6 h. The resulting powders were washed three times with acetone and de-ionized water and dried for a day in an oven at 70 °C. Finally, FTIR spectra were used to confirm the bioactivity of each specimen.

## 3. Results

**Figure 1:**
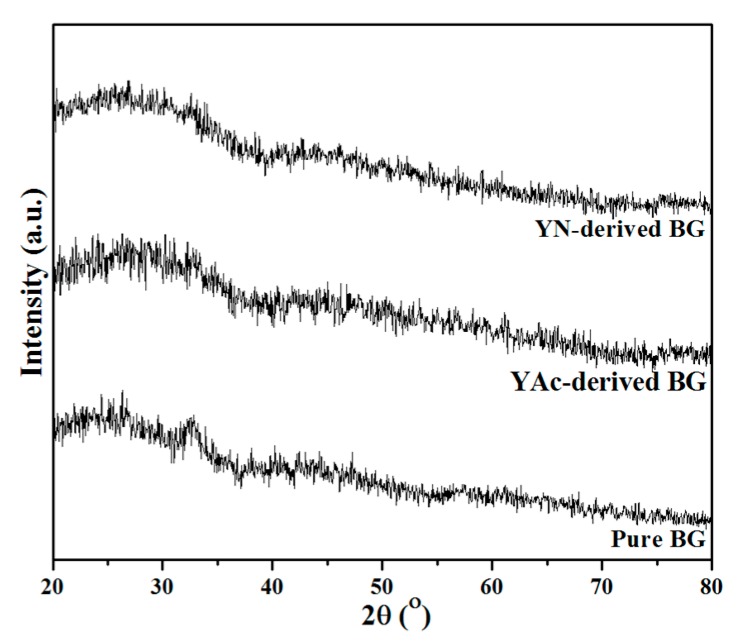
XRD patterns of pure BG powder, YAc-derived BG powder and YN-derived BG powder.

Figure 1 shows the XRD patterns of pure and Y-doped BG powders prepared using SP. First, for the pure BG powder, no distinct diffraction peaks can be observed from the XRD pattern with only a broad band existing between 20° to 40°. The result suggests that the structure of pure BG powder is amorphous. Meanwhile, both Y-doped BG powders from YAc and YN precursors show similar XRD patterns as the pure BG powder, indicating that all spray-pyrolyzed BG and Y-doped BG powders were synthesized successfully with a glassy phase.

**Figure 2:**
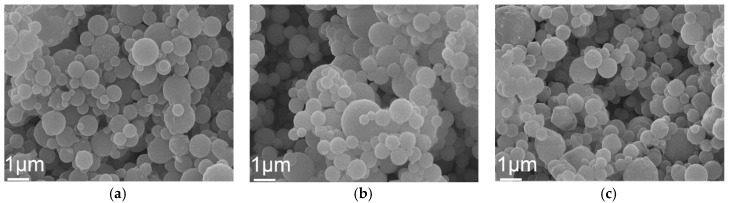
SEM micrographs of (**a**) pure BG powder, (**b**) YAc, and (**c**) YN derived Y-doped BG powders.

For the observation of surface morphologies and particle sizes, Figure 2 shows the SEM images of pure and Y-doped BG powders. It can be seen from Figure 2a that the pure BG powder exhibited a spherical morphology for all particles, which is the typical particle morphology for the spray pyrolysis process [21]. Meanwhile, it can also be seen from Figure 2b,c that both Y-doped BG powders from YAc and YN precursors have a similar surface morphology. In addition, statistical measurements of particle sizes of all spray pyrolyzed BG powders were calculated based on the SEM images. The measurements show that the average particle size of pure BG powder was 0.99 ± 0.40 µm, while Y-doped BG powders from YAc and YN salts were 0.94 ± 0.39 and 0.89 ± 0.34 µm, respectively. On the other hand, the BET data showed that the specific surface areas of pure BG powder, YAc derived Y-doped BG powder, and YN-derived Y-doped BG powder were 4.65 ± 0.07, 4.42 ± 0.08, and 10.08 ± 0.12 m^2^/g, respectively, which indicates the YN-derived Y-doped BG powder exhibited the highest specific surface area compared with the pure BG powder and YAc-derived Y-doped BG powder.

**Figure 3:**
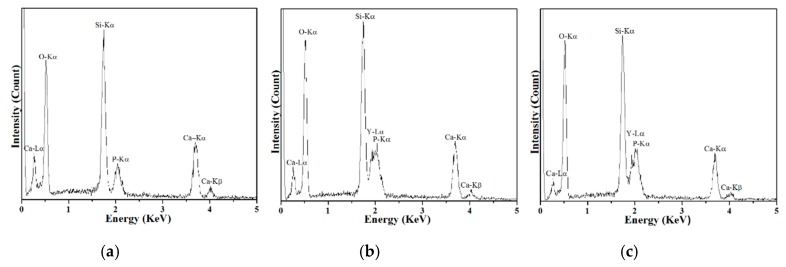
EDS spectra of (**a**) pure BG powder, (**b**) YAc, and (**c**) YN derived Y-doped BG powders.

Next, the compositional analysis of pure and Y-doped BG powders was carried out using EDS and the resulting spectra are shown in Figure 3. Excluding the peak at 0.275 eV, which corresponds to the C-Kα_1_ edge from the carbon paste, all BG powders showed peaks of O-Kα_1_, Si-Kα_1_, P-Kα_1_, Ca-Kα_1_, and Ca -Kβ_1_ at 0.519, 1.746, 2.039, 3.670, and 4.007 KeV, respectively. Moreover, Y-Kα_1_ and Y-Kβ_1_ edges were observed from both YAc- and YN-derived Y-doped BG powders. In addition, Si, Ca, and P concentrations were computed to be 58.35 ± 1.56, 31.33 ± 1.17, and 10.31 ± 0.76 at% for the pure BG powder; meanwhile, Si, Ca, P, and Y concentrations were 59.58 ± 4.55, 24.12 ± 2.50, 7.16 ± 0.76, and 9.14 ± 2.36 at% for the YAc-derived BG powder and 52.05 ± 1.83, 29.71 ± 0.97, 11.17 ± 1.06, and 7.05 ± 0.38 at% for the YN-derived BG powder. The results indicate that both pure and Y-doped BG powders were successfully synthesized with their given precursor concentrations. 

**Figure 4:**
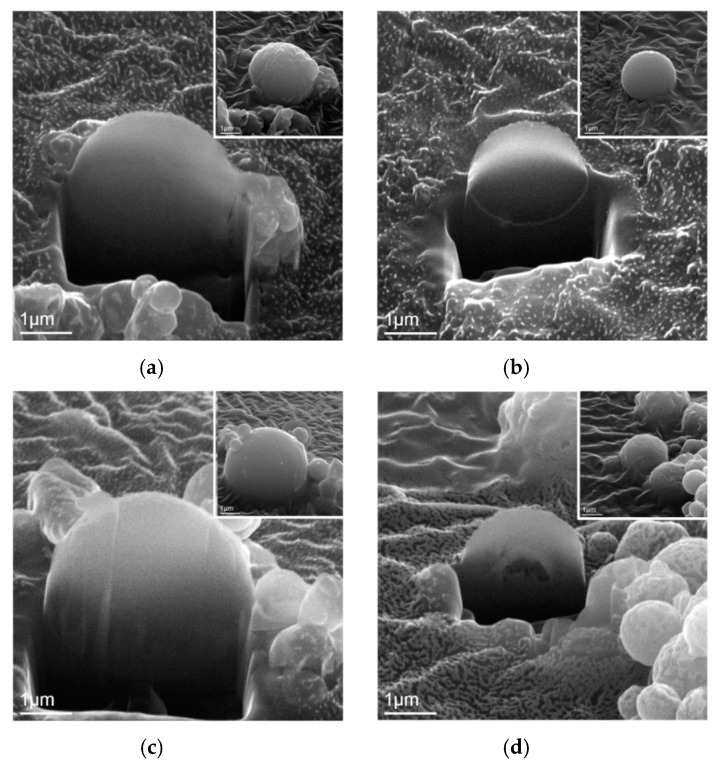
SEM images of focused ion beam-prepared cross-sectional solid particles of (**a**) pure BG powder, (**b**) YAc, and (**c**) YN derived Y-doped BG powders. (**d**) Cross sectional hollow particle of YN-derived Y-doped powder.

**Figure 5:**
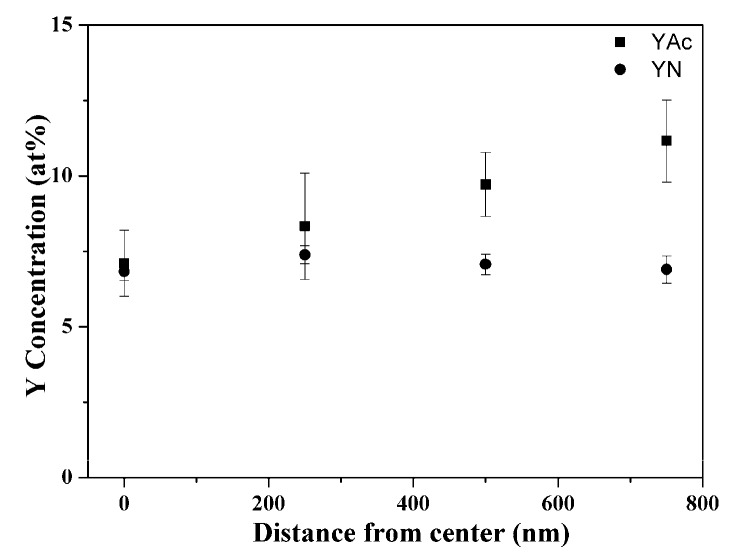
Mean Y concentration distributions of YAc- and YN-derived Y-doped BG powders.

Then, Figure 4 shows the SEM images of FIB-prepared cross-section specimens with insets of their as-prepared state. By examining more than 20 particles within each BG powder, it can be seen from Figure 4a that the pure BG particles exhibited an inner solid structure. In addition, YAc-derived Y-doped BG particles (Figure 4b) showed the same inner solid structure as the pure BG particles. However, for the YN-derived Y-doped BG particles, two morphologies of solid and hollow were observed as shown in Figure 4c,d. Furthermore, the local Y concentrations of each specimen were recorded and shown in Figure 5. The results show that for the YAc-derived powder (■), the Y concentration increased from the center (7.11 ± 1.09%) to the edge (11.16 ± 1.36%). Meanwhile the YN-derived powder (●) showed a consistent distribution of around 7% for the whole particle.

**Figure 6:**
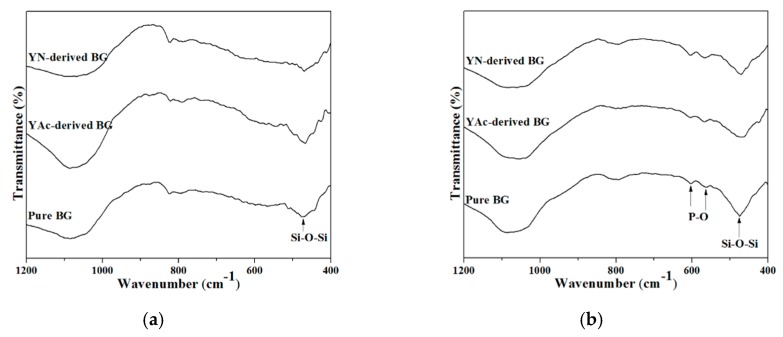
FTIR spectra of pure BG powder, and YAc- and YN-derived Y-doped BG powders: (**a**) before and (**b**) after immersion in SBF solution for 6 h.

At last, Figure 6 shows the FTIR spectra for examining the in vitro bioactivity. The results confirmed that both pure and Y-doped BG powders were bioactive due to the presence of the P–O bonding that formed. To determine the bioactivity of each specimen, a ratio of peak intensities were computed from the FTIR spectra [22,23], which will be discussed in the following paragraph.

## 4. Discussion

The first issue to discuss is the SP formation mechanism of the BG powders. Previous studies have demonstrated that there are two main formation mechanisms for the spray pyrolysis method, which are “one-particle-per-drop” and “gas-to-particle” [24]. Based on the observation of the SEM images shown in Figure 2, both pure and Y-doped BG powders exhibited a single morphology of spherical particles with no nanosized particles formed. This indicates that all spray pyrolyzed BG powders followed the formation mechanism of “one-particle-per-drop.” 

Next, the inner structures of all BG powders were examined using FIB-prepared cross-section specimens. During a spray pyrolysis system, the precursor droplets go through three stages of thermal treatments of solvent evaporation, solute decomposition, and particle calcination to form the final particles. Factors influencing the inner structure are suggested to be evaporation rates of the solvent, solubilities, and melting temperature of precursors [25,26]. For the pure BG powders, the inner solid structure was observed (Figure 4a) due to the high solubilities and high melting temperatures of precursors, the precursor droplets followed the “volume precipitation” mechanism, which precipitated homogenously during the SP [27]. By adding YAc, which also has a high melting temperature of 285 °C [28] into the precursor solution, it follows the same formation mechanism as pure BG powders and hence results in a solid structure (Figure 4b). However, for the precursor of YN, a low melting temperature of 88 °C [29] will let the solutes on the surface of the droplets melt during the solvent evaporation stage and trap the solvent in the center [27]. Thus, a solid shell will be formed and result in the hollow particle (Figure 4d). Meanwhile, the surface area of the YN-derived Y-doped BG powder was about two times higher than the other powders, verifying its inner structure was hollow.

Next, the local Y distributions in Y-doped BG powders were discussed. Considering the movement of ions may be assisted by the diffusion of water, the mobility of ions can therefore be related to the solubilities of the precursor. In this study, solubilities of YAc and YN precursors in water were 100 and 1550 g/L, respectively, at around room temperature [30,31]. In this case, the acetate groups of the YAc precursor dissolved better in the solution, leading Y ions to distribute closer to the droplet surface. In contrast, owing to the high solubility of the YN precursor, the ions will distribute more evenly throughout the particle. During the calcination stage, Y will precipitate simultaneously in the whole particle. YN-derived Y-doped BG particles will therefore have local Y distribution dispersed homogenously in the whole particle. 

At last, the in vitro bioactivity tests were carried out and examined using FTIR spectra. To determine the bioactivity of each specimen, a ratio of peak intensities, I_1_/I_2_, were computed from the FTIR spectra [22,23], where I_1_ is the intensity of P–O bending vibration around 567cm^−1^ [32,33] and I_2_ is the intensity of the Si–O–Si bending vibration at 482 cm^−1^ [34,35]. This value correlates to the amount of hydroxyapatite (HA) formed, i.e. the higher the I_1_/I_2_ ratio, the larger amount of HA was formed which represents higher bioactivity. Following Figure 6, the resulting I_1_/I_2_ values of the pure BG powder, and YAc- and YN-derived Y-doped BG powders were computed to be 0.32, 0.44, and 0.53, respectively, indicating the order of bioactivity was pure BG powder < YAc-derived Y-doped BG powder < YN-derived Y-doped BG powder, similar to the measured surface areas. 

## 5. Conclusions

In this study, both pure and Y-doped BG powders were successfully synthesized using spray pyrolysis method. The composition analysis shows that the local Y distributions were controlled by the addition of YAc and YN, while the particles’ morphologies and surface areas were also observed. At last, both Y-doped BG powders were confirmed to be bioactive with the formation of HA after immersing in the SBF for 6 h. Thus, it can be concluded that Y-doped BG powders can be a potential candidate for the effective treatment of cancerous cells in selective internal radiotherapy applications.
